# Antibiotics and probiotics-induced effects on the total fatty acid composition of feces in a rat model

**DOI:** 10.1038/s41598-024-57046-6

**Published:** 2024-03-19

**Authors:** Tamás Marosvölgyi, Kitti Mintál, Nelli Farkas, Zoltán Sipos, Lilla Makszin, Éva Szabó, Attila Tóth, Béla Kocsis, Krisztina Kovács, Edina Hormay, László Lénárd, Zoltán Karádi, Anita Bufa

**Affiliations:** 1 Institute of Bioanalysis, Medical School, University of Pécs, Pécs, 7624 Hungary; 2 Institute of Physiology, Medical School, University of Pécs, Pécs, 7624 Hungary; 3 Medical and Engineering Multidisciplinary Cellular Bioimpedance Research Group, Szentágothai Research Centre, University of Pécs, Pécs, 7624 Hungary; 4 Department of Biochemistry and Medical Chemistry, Medical School, University of Pécs, Pécs, 7624 Hungary; 5 Department of Medical Microbiology and Immunology, Medical School, University of Pécs, Pécs, 7624 Hungary

**Keywords:** Lipids, Nutrition

## Abstract

Fatty acids (FAs) play important roles as membrane components and signal transduction molecules. Changes in short chain FA (SCFA) composition are associated with gut microbiota modifications. However, the effect of bacteria-driven changes on the detailed FA spectrum has not been explored yet. We investigated the effect of antibiotics (ABx) and/or probiotics, in four treatment groups on rat stool FA composition. Principal component analysis indicated that the chromatogram profiles of the treatment groups differ, which was also observed at different time points. Linear mixed effects models showed that in the parameters compared (sampling times, treatments. and their interactions), both the weight percentage and the concentration of FAs were affected by ABx and probiotic administration. This study found that the gut microbiome defines *trans* and branched saturated FAs, most saturated FAs, and unsaturated FAs with less carbon atoms. These results are among the first ones to demonstrate the restoring effects of a probiotic mixture on a substantial part of the altered total FA spectrum, and also revealed a previously unknown relationship between gut bacteria and a larger group of FAs. These findings suggest that intestinal bacteria produce not only SCFAs but also other FAs that may affect the host’s physiological processes.

## Introduction

Fatty acids (FA) are important constituents of all multicellular organisms, primarily as components of the phospholipid bilayers of the cell membranes, and they also have an essential role in normal growth and development. In general, they have a hydrocarbon chain with a carboxyl group at one end and a methyl group at the other with chain lengths ranging from 2 and close to 30. These chains may be presented in two main forms: saturated fatty acids (SAT) are loaded with hydrogen containing only C–C single bonds and unsaturated fatty acids which contain at least one double bond^1,2^. Both saturated and unsaturated FAs are elementary nutrients and as components of triacylglycerols they also serve as major energy stores. Branched-chain fatty acids are synthesized by microorganisms (mainly bacteria) from branched-chain amino acids and therefore, they can be mainly found in bacterial membranes, or ruminant-derived lipids, e.g. milk or dairy products^3^. Depending on their various structures they have unique biological properties and as part of different lipid classes they play central role of diverse physiological processes including lipid and energy metabolism, endothelial functions, homeostatic and inflammatory responses along with having central role in functioning the central nervous system (CNS)^1,4–7^.

The intestinal microbiome is a collection of highly diverse microbial communities that provide the host with a broad range of fundamental functions, including protection from pathogens, impact the host physiology via metabolites and shaping the activity of the adaptive immune system. The relationship between FAs and the gut microbiome is thought to be bidirectional: the dietary FA intake can influence the type and abundance of gut microbes, while the microbiome also affects the metabolism and absorption of dietary fatty acids^8,9^. Previous studies have shown that diets differing only in FA composition (high SAT, high monounsaturated fatty acid (MUFA) or high polyunsaturated fatty acid (PUFA) content) are able to efficiently influence the distribution of intestinal microbial populations (as phylum and family levels)^10,11^.

While the effect of diets containing different types of FAs on the microbiota has been extensively studied^11–14^, wide-ranging research on the effects of gut microbes on fatty acids are comparatively scarce. Changes in the microbiome can be caused by alterations in the FA profile, however, these studies focus mostly on the short-chain fatty acids (SCFAs)^11^. Intestinal bacteria could also be able to influence the host FA profile in different tissues since some commensal bacteria can generate bioactive isomers: for instance, conjugated linoleic acid can be produced by *Lactobacilli*^9,15^ and *Bifidobacteria*^15,16^ or can produce *trans* fatty acids, such as vaccenic acid^15,17^. Furthermore, altered gut microbiota, in particular the genera *Prevotella*, *Lactobacillus* and *Alistipes* can increase capacity to produce saturated long chain FAs and thus affect gut motility^18^. During fermentation of nondigestible carbohydrates, the gastrointestinal (GI) microbiota produces a wide range of secondary metabolites, among others SCFAs (acetate, propionate, and butyrate)^19,20^. However, protein fermentation can also contribute to the SCFAs production, but these are mostly branched-chain FAs such as isobutyrate, 2-methylbutyrate and isovalerate^8–11,21,22^. SCFAs can have diverse functions, like providing energy for both the microbiota and the enterocytes^23^. In addition, SCFAs could enter the circulation and exert their additional health-promoting benefits to the host acting as secondary messengers that regulate gene expression and stimulate gut peptides and hormones^20,22^ and they are able to cross the blood–brain barrier^23^. The effect of gut microbiota via the SCFAs is therefore not restricted to the GI tract. Recent research showed that changes in gut microbiota could affect the host physiological, behavioral, and cognitive functions^24–26^, and therefore there is an emerging need to understand the complex effects of the relationship between the specific composition and activity of gut microbiota (at phyla, genus or species level) and the CNS of the host.

The composition and function of the GI microbiota is profoundly influenced by a variety of environmental factors such as genetics of the host, age, smoking, diet, exercise, mode of delivery, geographic location, synthetic chemicals, and antibiotic intake^27,28^. Furthermore, there are other modulations which can be carried out via dietary interventions, antibiotics (ABx) administration, supplementation of probiotics and fecal microbiota transplantation^29^, which all have a capacity to highly modulate the composition of the gut microbiota. Both prebiotics (non-digestible food ingredients which can selectively stimulate the growth and/or activity of beneficial bacteria), probiotics (non-pathogenic microbes which have beneficial effects on host) and ABx affect the composition of the gut microbiota without directly affecting the FAs in the gut^30,31^. Therefore, the changes in host FAs that occur because of the above-mentioned factors are likely to be directly related to the microbiome. These interventions could selectively modulate the microbiome composition depending on the combination of probiotic or prebiotic or the ABx class, dose, and the period of exposure^30,32^. These microbiome influencing factors provide an opportunity to study the aspects of the modifications of the GI bacteria and the influence of these to the host physiology including immune system, metabolism pathways, hormone activation and behavior^33^. Since the literature has mainly focused on the effect of ABx and probiotic treatment on the concentration of fecal SCFAs^34–36^, but to the best of our knowledge the amount of longer chain and *trans* isomers in feces has not yet been investigated, our aim in the present study was to determine the total FA spectrum. Therefore, the rats were given probiotics, ABx and a combination of these to carry out bacterial alterations without modifying the FA profile of the diet. Our further aim was to compare the time course effects of the different treatments on the total FA spectrum of feces (relative and absolute concentrations of short chain, branched chain, saturated, *trans* and cis mono- and polyunsaturated FAs) via gut bacteria.

## Results

### Changes in the fecal fatty acid composition

**Figure 1:**
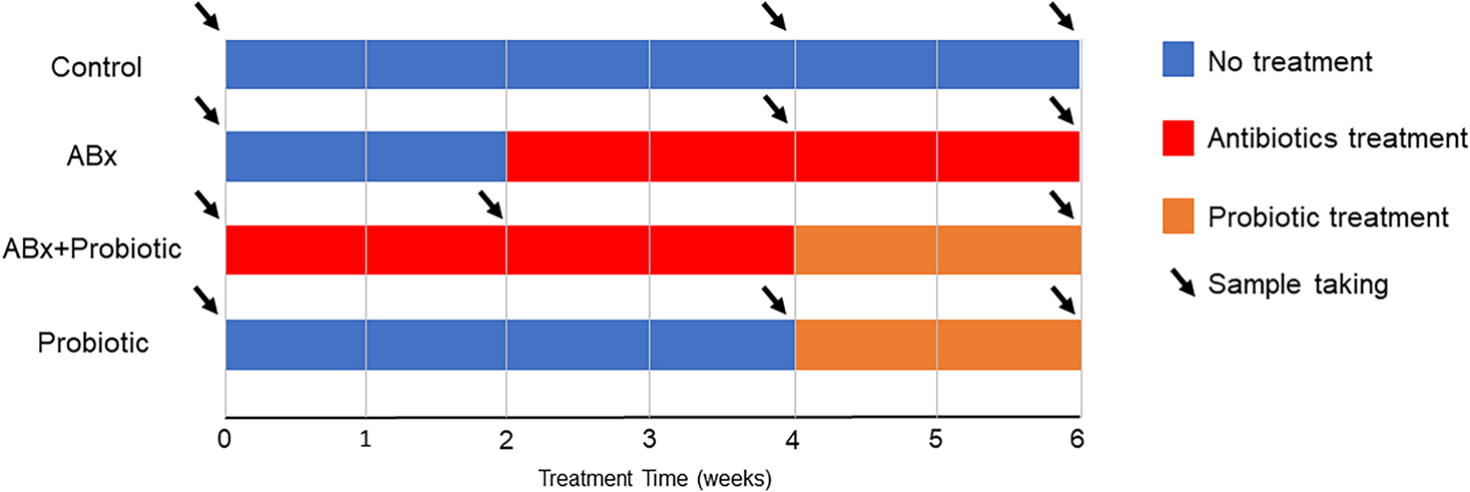
Experimental arrangement of the treatment groups to demonstrate the time and duration of antibiotics and probiotic treatments. ABx: broad-spectrum antibiotics treated group, ABx + Probiotic: broad-spectrum antibiotics- and then probiotic treated group, Probiotic: probiotic treated group, Control: control group without any treatment. Sampling was done three times in each group at the indicated times. First sampling in each group was done at start of the experiment, before treatment was started (baseline data).

**Figure 2:**
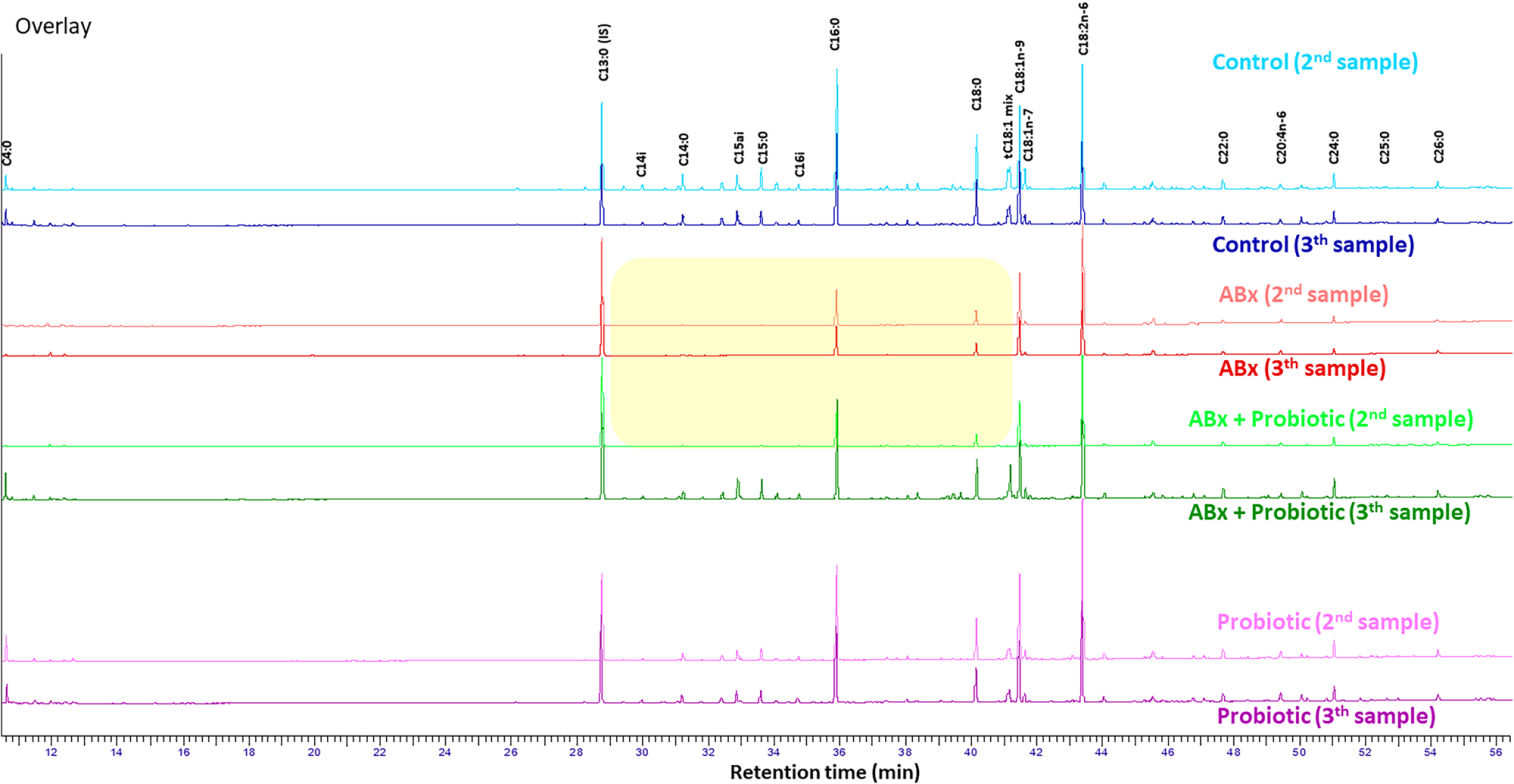
Representative GC chromatograms (from 1–1 identical rat per group) of rat feces between C4:0 and C26:0 fatty acids during the treatments*.* Chromatograms of fecal samples were graphically normalized to the peak height of the internal standard C13:0.

The fatty acid composition of fecal samples from four different study groups (Control, ABx, ABx followed by Probiotics, Probiotics) was analyzed before (1st sample), during (2nd sample) and at the end of the treatments (3rd sample), at a total of three time points per group (Fig. 1). While analyzing the effect of the different treatments on the fatty acid methylesters (FAMEs) (C4:0 to C26:0) in the fecal samples, we observed large differences between the chromatograms of the second and the third samplings (Fig. 2). Most of the major components of fatty acids (C16:0, C18:0, C18:1n-9, C18:2n-6) remained detectable in each treatment group, however, these values were lower in the ABx-treated groups (ABx 2nd and 3rd sample, ABx + probiotic 2nd sample) compared to the ABx-untreated groups (control 2nd and 3rd sample, ABx + Probiotic 3rd sample, Probiotic 2nd and 3rd sample). Furthermore, many saturated, branched-chain saturated, monounsaturated and *trans* fatty acids between chain length C14:0-C20:0 were reduced to the limit of detection as a result of ABx treatment (in Fig. 2 shown with yellow background).

**Figure 3:**
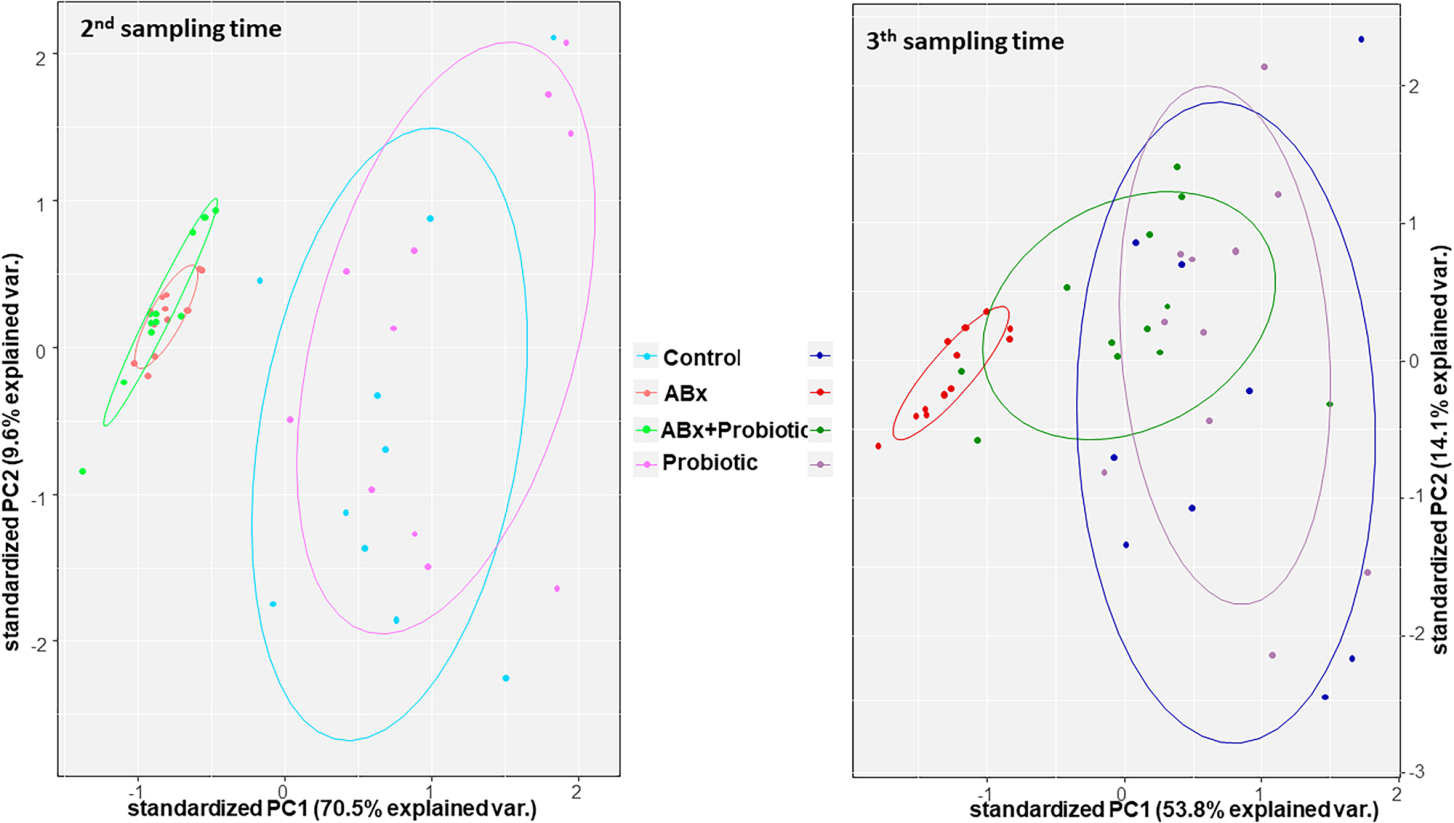
Plots of the two main components (PC1 and PC2) of the principal component analyses. The graphs represent the second and third sampling time, which were made based on the areas under the chromatogram curve data. The colors of the groups correspond to those in the second figure, and the dots represents the rat samples.

A principal component analysis (PCA) was applied to confirm the different characteristics of these chromatograms. This analysis showed that the areas under the chromatogram curve of FAs differed because of the different treatments. The two main principal components (PC1 and PC2) explain 80.1% of the variances of the differences in the chromatograms in the case of the second sampling time. The PCA model clearly separates the samples into two groups. The first group consists of the ABx and the ABx + Probiotic; whereas, the Control and the Probiotic groups are in the second. Although PC1 and PC2 explain only 67.9% of the variances of the differences between the chromatograms in the case of the third sampling time, we can see that the PCA scores of the ABx + Probiotic group are closer to those of the Control and Probiotic groups than those of the ABx group. Whereas the ABx group remains separate from the other groups (Fig. 3).

### Mixed model analysis

A nested linear mixed random effect model was used to investigate the differences between the concentrations and the weight percentage ratios of the FA components.

The results showed that the main categories of the examined FAs exhibited substantial differences in the parameters we compared (time points of sample taking, treatments and their interactions) both in the analysis of the concentration and weight percentage ratio for total FA content (Table 1). In addition, data of each examined FAs were also analyzed, the results of which are provided in Supplementary Table S1, S2 and S3. There were significant differences between the compared parameters for most FA concentrations tested, indicating that the gut microbiota affected the values of these FAs.

**Table 1:** Results of the mixed effect models of the main fatty acids categories.

Fatty acid groups	The nested linear mixed effect model					
	*p* value for concentrations			*p* value for weight %		
	Time point	Treatment	Time point x treatment	Time point	Treatment	Time point x treatment
Total FA	1.56 × 10^–6^	1.10 × 10^–6^	6.91 × 10^–7^			
SAT	2.90 × 10^–8^	1.21 × 10^–13^	3.27 × 10^–10^	2.81 × 10^–3^	2.20 × 10^–11^	4.46 × 10^–7^
*ShortSAT*	5.12 × 10^–13^	6.31 × 10^–10^	2.54 × 10^–10^	6.34 × 10^–7^	7.41 × 10^–8^	2.11 × 10^–3^
Transbranched	1.03 × 10^–12^	1.27 × 10^–15^	< 2.20 × 10^–16^	1.54 × 10^–6^	2.82 × 10^–8^	2.81 × 10^–11^
*Branched sat*	3.58 × 10^–11^	< 2.20 × 10^–16^	< 2.20 × 10^–16^	1.24 × 10^–5^	< 2.2 × 10^–16^	1.07 × 10^–13^
*Trans*	5.82 × 10^–7^	< 2.20 × 10^–16^	3.55 × 10^–14^	2.53 × 10^–3^	7.60 × 10^–13^	8.06 × 10^–8^
cMUFA	8.44 × 10^–5^	8.73 × 10^–7^	5.80 × 10^–4^	0.163	1.14 × 10^–6^	6.73 × 10^–3^
PUFA	2.04 × 10^–2^	3.43 × 10^–2^	0.218	1.03 × 10^–4^	< 2.20 × 10^–16^	2.10 × 10^–8^
*n-6 PUFA*	1.94 × 10^–2^	3.64 × 10^–2^	0.221	1.38 × 10^–4^	3.26 × 10^–16^	2.23 × 10^–8^
*n-3 PUFA*	4.85 × 10^–2^	1.53 × 10^–2^	0.214	1.58 × 10^–4^	1.78 × 10^–14^	9.28 × 10^–6^
LCPUFA	2.57 × 10^–3^	5.50 × 10^–4^	0.127	4.38 × 10^–4^	4.49 × 10^–6^	1.13 × 10^–6^
*n-6 LCPUFA*	2.62 × 10^–3^	5.43 × 10^–4^	0.128	4.27 × 10^–4^	4.64 × 10^–6^	1.09 × 10^–6^
*n-3 LCPUFA*	0.576	0.308	0.797	0.916	0.560	0.915

*p*-values of the mixed effects models (*p* < 0.05). *Abbreviations denote*: saturated fatty acid (SAT): ShortSAT + C9:0 + C10:0 + C11:0 + C12:0 + C14:0 + C15:0 + C16:0 + C17:0 + C18:0 + C20:0 + C22:0 + C23:0 + C24:0 + C25:0 + C26:0; short saturated fatty acid (ShortSAT): 4:0 + C5:0 + C6:0 + C7:0 + C8:0; Transbranched: Branched Sat + TRANS + (tC16:1n-7 + C17i) + (tC17:1n-7 + C18i); *Trans*: sum of tC18:1 isomers; Branched saturated fatty acids (Branched Sat): C13ai + C14i + C15i + C15ai + C16i; cis monounsaturated fatty acid (cMUFA): C13:1n-1 + C14:1n-5 + C16:1n-9 + C16:1n-7 + C18:1n-9 + C18:1n-7 + C20:1n-9 + C22:1n-9; polyunsaturated fatty acid (PUFA): n-3 PUFA + n-6 PUFA; n-3 PUFA: C18:3n-3 + n-3 LCPUFA, n-6 PUFA: C18:2n-6 + n-6 LCPUFA long-chain polyunsaturated fatty acid (LCPUFA); n-3 LCPUFA + n-6 LCPUFA; n-3 LCPUFA: C20:5n-3; n-6 LCPUFA: C20:2n-6 + C20:3n-6 + C20:4n-6 + C22:4n-6 + C22:5n-6.

### Saturated fatty acids (SAT)

The time points, the treatments and their interaction demonstrated significant differences, indicating that both ABx and subsequent probiotic treatment influenced the concentration (Supplementary Figure S1) and weight percentage ratio (Supplementary Figure S2) of this category (Table 1) suggesting, that GI microbiome played a major role in the production of most of the FAs in this category. Except for C25:0, which only showed tendencies, there were significant differences in the absolute concentrations of FAs between C12:0 and C26:0 between time points, treatment groups and their interaction. This indicates that the treatments were effective in modifying the concentrations of these FAs, in particular, the ABx treatment effectively reduced them, which was subsequently restored after the use of probiotic mixture (Fig. 2 and Supplementary Table S2). For C10:0, there was a significant difference in both absolute and relative concentrations between the time points and treatments, but not in their interaction. This indicates that the ABx treatment reduced the absolute and relative concentration, however, the increase in concentration was not due to the probiotic treatment.

The weight percentage ratios of C14:0, C15:0, C17:0 and C18:0 significantly differed between the time points and between the treatment groups and there was a significant interaction too. Thus, the ABx treatment decreased the weight percentage ratio of these FAs, which the probiotic treatment effectively restored. In the case of weight percent ratio of C26:0, both the time point and the treatment significantly differed, whereas their interaction not. These observed changes were triggered by the administration of the ABx and not the probiotics. (Fig. 1 and Supplementary Table S1-S3).

### Short saturated fatty acids (short SATs)

The short saturated fatty acids (short SATs) is a subcategory of SAT, including five FAs (C4:0, C5:0, C6:0, C7:0, C8:0) in this study. This subcategory also presented significant differences in time points, treatments and their interaction, indicating that both FA concentrations (Supplementary Figure S1) and weight percent ratios (Supplementary Figure S2) were modified by the different treatments (Table 1). The absolute concentrations of C4:0, C5:0 and C7:0 differed significantly between the time points and treatment groups with a significant interaction between them. This indicates that ABx treatment effectively reduced the concentration, which was successfully restored after probiotic treatment (Fig. 2 and Supplementary Table S1, S2). The concentrations of C6:0 and C8:0 significantly differed between both the time point and treatment, whereas their interaction not, so ABx treatment reduced their concentration, but the increase in the concentration was not due to the probiotic treatment.

Significant differences in weight percent between time points, treatments and their interaction were only found for C4:0, while for C6:0 and C8:0 only treatment caused a significant change (Fig. 2 and Supplementary Table S1, S3). In the case of C5:0, both the time point and the treatment significantly differed, whereas, their interaction did not. These observed changes were triggered by the administration of the ABx and not the probiotics. In the case of C6:0 and C8:0, only the treatment showed a significant effect meaning that the ABx treatment reduced their weight percentage ratios, but did not change after the probiotic treatment.

### Trans and branched fatty acids

This main category consists of three coelutions (*t*C17:1n-7 + C18i, *t*C16:1n-7 + C17i and *t*C18-1mix) and five branched-chain saturated FAs, which showed large differences. There were significant differences in both concentration (Supplementary Figure S1) and weight percent values (Supplementary Figure S2) between time points, treatments and their interactions, so both the ABx and probiotic treatment effectively altered them (Table 1).

### Branched saturated fatty acids (branched SATs)

In this subcategory, all the examined branched SATs (C13ai, C14i, C15i, C15ai and C16i) showed strong significant differences between sampling times, treatments and their interactions in both concentration (Supplementary Figure S1) and weight percentage ratio (Supplementary Figure S2) for total FA. These results suggest that altered gut microbiota due to treatments were responsible for the observed alterations. The ABx treatment significantly decreased the levels of identified branched SATs, while after probiotic administration these values returned (Fig. 2 and Supplementary Table S1, S2 and S3).

### Trans fatty acids (TFAs)

The ABx treatment significantly decreased both the concentration (Supplementary Figure S1) and the weight percentage ratio of *t*C18:1 fatty acids (Supplementary Figure S2), but subsequent probiotic administration was able to increase these levels to the level before the treatments (Fig. 2 and Supplementary Table S1, S2 and S3).

### Cis monounsaturated fatty acids (cMUFAs)

In the cMUFAs almost all the parameters tested showed significant differences in both absolute concentration (Supplementary Figure S1) and weight percent ratio (Supplementary Figure S2) indicating that ABx treatment and probiotic administration were the main factors influencing these concentrations (Table 1). In this category we examined 8 cMUFAs (C13:1n-1, C14:1n-5, C16:1n-9, C16:1n-7, C18:1n-9, C18:1n-7, C20:1n-9 and C22:1n-9). Except for C22:1n-9, which showed only tendencies, most of these cMUFAs revealed significant differences in concentration values between time points, treatments, and their interactions. Thus ABx treatment significantly decreased the concentration levels, while the administration of the probiotics restored them (Fig. 2 and Supplementary Table S1, S2).

However, when evaluating the weight percentage ratio, we found significant effects of time and interaction between time and treatments for only three fatty acids (C13:1n-1, C14:1n-5 and C18:1n-9 FAs). The ABx treatment decreased the weight percent ratio of C13:1n-1 and C14:1n-5, and elevated that of the C18:1n-9. On the other hand, probiotic treatment recovered the weight percent ratios for all three FAs to the original state. (Fig. 2 and Supplementary Table S1, S3).

### Polyunsaturated fatty acids (PUFAs)

Significant differences were observed in both concentration (Supplementary Figure S1) and weight percent ratio (Supplementary Figure S2) of PUFAs indicating that the FAs of this category were modified by the different treatments (Table 1, Supplementary Table S1, S2 and S3). The PUFAs were splitted in two subcategories: n-3 PUFAs (α-linolenic acid (C18:3n-3, ALA) and C20:5n-3) and n-6 PUFAs (C18:2n-6, C20:2n-6, C20:3n-6, C20:4n-6, C22:4n-6 and C22:5n-6) (Supplementary Table S1). In the weight percent ratio, each investigated parameter showed significant differences, thus ABx treatment decreased levels of both n-3 and n-6 PUFAs, which could successfully recovered after the use of probiotic treatment (Supplementary Figure S2, Supplementary Table S3). In contrast, concentration levels were significantly affected by both time point and treatment, but their interaction not. This indicates that the ABx treatment reduced their concentration, but their increase over time was not dependent on the probiotic treatment (Supplementary Figure S1). A further subcategory within PUFAs is the long chain polyunsaturated fatty acids (LCPUFAs) that also can be divided in 2 subgroups: n-6 LCPUFAs (C20:2n-6, C20:3n-6, C20:4n-6, C22:4n-6 and C22:5n-6) and n-3 LCPUFAs (C20:5n-3) (Supplementary Table S1). The LCPUFAs subcategory and the n-6 LCPUFAs subgroup showed similar results as the PUFAs category in both concentration and weight percent ratios, but there were no significant differences in the n-3 LCPUFAs subgroup (Table 1, Supplementary Figure S1-S2).

As C20:5n-3 did not show significant differences, it can be concluded that C18:3n-3 was the determining FA in the n-3 PUFAs category, showing the same significances in the concentration level as its group, however, in the weight percent ratio differences were noted in the interactions of time points and treatments. Among the n-6 PUFAs subcategory, only two FAs (C20:2n-6 and C20:3n-6) presented significant differences in time points, treatments and their interactions in the concentration levels. In those cases, decreased concentration levels were measured after ABx treatment, and probiotic mixture was able to restore it. On the other hand, weight percent ratios of C18:2n-6 and C20:4n-6 were increased after ABx treatment and the following probiotic administration could efficiently restore these altered states.

### Fatty acid composition of the pellets

**Figure 4:**
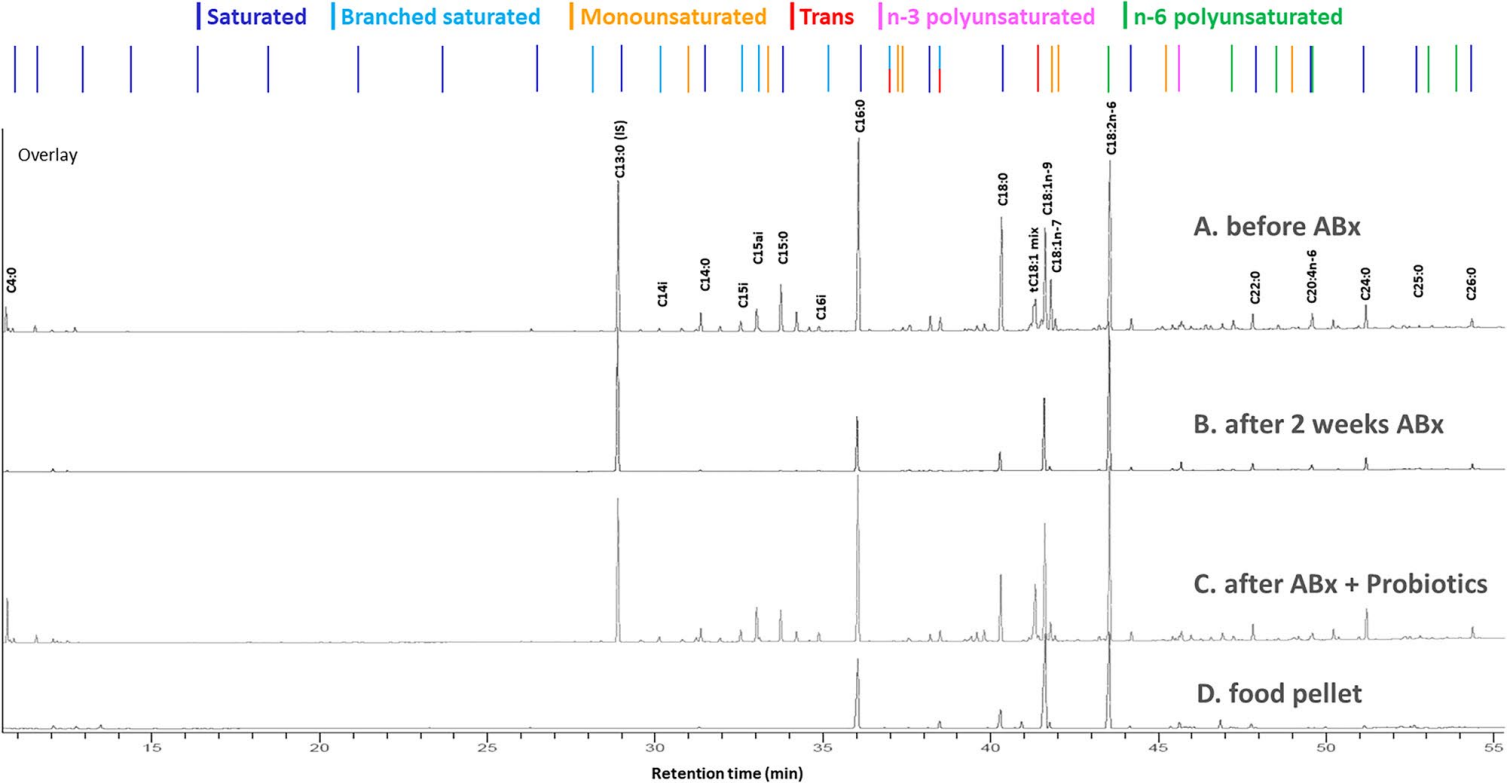
Changes in the chromatograms of fatty acids between C4:0 and C26:0 in the feces of a treated rat from the ABx + Probiotic group at baseline (**A**), after two weeks of antibiotic treatment (**B**), then after probiotic treatment (**C**) and the chromatogram of dry rodent food (**D**). In the upper part of the figure, the order of the individual fatty acids is indicated by colors corresponding to the different fatty acids families (dark blue: saturated, light blue: branched saturated, orange: monounsaturated, red: trans, pink: n-3 polyunsaturated, and green: n-6 polyunsaturated fatty acids). The chromatograms of fecal samples were graphically normalized to the peak height of the internal standard C13:0.

Since the examined FA composition of the feed was not fully provided, we specified the comprehensive FA composition of the rodent chow (Supplementary Table S4) and compared it with the treatment effects, which is illustrated in Fig. 4. The analysis revealed that out of the examined FAs the MUFAs were present in the highest quantity in the rat feed, alongside PUFAs and SATs. In addition, n-6 PUFAs were more dominant than n-3 PUFAs, and, in these categories, the most prevalent fatty acids were C18:2n-6 and C18:3n-3. Among SATs, the highest quantities observed in the rats' food were for C16:0 and C18:0. Short SATs were present only in a small proportion in the food. No Branched SATs were found in the animal feed. Among MUFAs, C18:1n-9 was the most abundant FA in the pellet. Furthermore, within the category of *TFA*s, *t*C18:1 fatty acids were present, but in a small amount.

The obtained results indicate that the FAs present in the rat feces and their changes resulting from the treatments were not influenced by the FA composition of the feed.

## Discussion

In this series of experiments, we induced a change in the GI microbiome with ABx and probiotic treatment to examine changes in fecal FAs. To the best of our knowledge, the present paper is the first report on the role of gut bacteria in a detailed fecal FA spectrum in adult rats.

There is increasing evidence that GI microbiome has a huge impact on regulating fat storage and energy metabolism, helps to absorb minerals and influences FA production^9,16,18^. Moreover, it plays a crucial role in regulating immune homeostasis, as well as CNS-associated neurophysiological and -psychological functions and behavior^37,38^. It is worth noting that not only just SCFAs are produced exclusively by bacteria, but also concentration of some FAs with a longer, odd-numbered carbon atoms, such as C15:0 and C17:0, are also affected by the microbiome^18^. Furthermore, some microbial species are able to metabolize C18:2n-6 and C18:3n-3^9^ and convert them to conjugated linoleic acids and other *trans* fatty acids such as *trans* vaccenic acid (C18:1n-7*t*)^17^.

As the composition of the microbiome can be influenced by early colonization and a number of other environmental factors^27,28^, the present study included rats born at the same time in the same place with the same genetic background, consuming the same food, and were randomly assigned to groups before the treatments. Thus, we assume that microbiota composition did not differ significantly between the groups of animals before the start of the experiment.

To investigate the effect of the microbiome on FA production we selected an ABx treated animal model. This wide spectrum, high dose treatment was able to deplete some gut bacterial community^39,40^, providing us a good opportunity to examine the bacterial effect on the whole FA spectrum in adulthood. ABx treatment can provoke extensive dysbiosis and the disappearance of SCFAs confirm the vanishing of the gut bacteria^34,36^. It is well known that fecal SCFAs are produced by the gut microbiota^8–11^, but our current results suggest that many other important saturated and unsaturated FAs across the entire FA spectrum are also produced by GI bacteria.

Previous studies have shown, that gut microbes play major role in the production of short SATs, of which C4:0 and C5:0 are the most important and play a critical role in both peripheral and central functions being involved in anti-inflammatory effects and playing a beneficial role in a variety of diseases^41,42^. Within the longer chain SATs, our finding revealed that the majority of these FAs are produced by intestinal bacteria, and not just those with odd-numbered carbon atoms as would be assumed based on former studies. Interestingly, C16:0 also showed reduced concentration after ABx treatment despite being one of the main FA components of the rodent's chow. The amount of all determined branched SATs was also significantly reduced after ABx treatment, confirming the finding of a recent study that branched saturated FAs originate from bacterial degradation of proteins^43^ and that lack of bacteria may generate the absence of branched SATs, that may be present in several metabolic disorders^44^. Although previous studies have shown that bacteria are the primary producers of branched-chain fatty acids, the extent of their absorption in the human body and their fate remains unclear. These fatty acids are present in various human tissues in a low concentration and may have metabolic health benefits, including lipid-lowering, and anti-inflammatory properties^3,45^. However, extensive research is still needed to understand their mechanism of action.

The majority of the analyzed cMUFAs were decreased after ABx treatment suggesting that the GI microbiome is responsible for the production of these FAs. However, there is no data on whether cMUFAs produced in the gut has any effect on health, or whether cMUFAs in the gut are not due to dietary intake, i.e., produced by gut bacteria.

The investigated n-3 PUFAs and most of the n-6 PUFAs, such as C18:2n-6, which was present in higher amounts in the rodents’ chow, did not show a decrease in their concentration after ABx treatment, corroborating the common assumption that these FAs are greatly modulated by dietary intake^46,47^. Among the minor components, there was a slight reduction in the concentrations of C22:4n-6 and C22:5n-6 however, we assume that these differences are actually due to their low concentrations. Surprisingly, the concentrations of C20:2n-6 and C20:3n-6 were also influenced by gut bacteria. Although so far only a few studies have investigated the relationship between the microbiome and these n-6 PUFAs in rumen fluid, plasma, and hepatic cells^48–50^, we could demonstrate a similar but only partial relationship, that the microbiome is responsible for the production of C20:2n-6 and C20:3n-6 in the gut, and not the dietary intake.

In our study, we determined not only absolute but also relative concentration changes, Our results showed, that in the majority of FAs both concentrations decreased after two weeks of ABx treatment. However, for weight percent, some FAs required a relative increase, such as in our case C20:4n-6 and C18:1n-9, while their absolute concentration also decreased after ABx treatment, so the contradiction between the two results is only apparent.

In this study, two weeks of broad-spectrum ABx treatment was sufficient to greatly reduce GI bacteria. Therefore, it would be important to prevent the adverse side effects of intensive ABx medication, including altered fecal FA distribution. We used our probiotic mixture because it contained bacterial populations with health-promoting effects, such as *Lactobacillus spp.,* most strains of which have great potential to reduce inflammation and alleviate ABx-induced GI side effects^51–53^, and *Bifidobacterium spp.,* which also have immunomodulatory effects and can be beneficial to the digestive and even the nervous system^52,54,55^. In addition, our aim was to restore the gut microbiota in a short time and to study the effect of this probiotic mixture on the detailed FA spectrum. As we hypothesized, probiotic administration was able to initiate the reestablishment of bacterial populations and promoted changes in the concentration of several FAs. Our results confirmed, that ABx treatment decreases the concentration of fecal SCFAs, whereas the following probiotic treatment (including *Lactobacillus* and *Bifidobacterium* strains) increases both the concentration of these FAs and fecal bacterial abundance and diversity^35^. However, our mixture was unable to restore the concentration of each FA to baseline. We hypothesized that in these cases (C6:0, C10:0, C11:0, C16:1n-9 and C16:1n-7 ) other bacterial genus species or additional microbial interactions may be involved, and accordingly, we confirmed that *Bifidobacterium spp*. and *Lactobacillus spp*., were clearly not responsible for the production of these FAs. Nevertheless, most of the FAs, whose concentrations had been altered by the microbiome depletion, returned to their initial state after probiotic administration, which confirmed that *Bifidobacterium spp*. and *Lactobacillus spp*. were able to induce the production of these FAs or perhaps they interacted with other bacteria to cause this change, which is a currently existing phenomenon^56^. Research lately has begun to explore the possible relationship between the microbiome and fecal metabolites, such as FAs. A recent study showed a strong positive correlation between *Lactobacillus* strains and fecal C18:2n-6 and C12:0 in a rat model of short bowel syndrome, while other bacterial strains (e.g. *Ruminococcus*) showed a negative correlation^57^. Based on our results, we hypothesized that our probiotic mixture had a crucial role in the production of branched SATs, most SATs and even several MUFAs, and PUFAs, and therefore, by administering these strains after ABx treatment, a significant part of the fecal FA spectrum could be restored. The fact, that the weight percent ratios were also restored means that the probiotic administration may have contributed to the changes in these proportions in the proper direction. Since all groups of animals were fed the same diet with the same FA composition, it can be assumed that any FAs whose concentrations changed during the treatments were produced by the gut bacteria and not derived from the diet.

The used microbiome alterations and the study design allowed us to determine the influence of the intestinal bacteria on the fecal FA composition, however, the present study has several strengths and limitations.

One strength of this paper is that the rats included in this study were of the same age and sex at the start of the study, so the confounding effects of age and sex were excluded, and the litter effect was also statistically corrected. Another strength is that, contrary to previous studies, not only SCFAs but the full spectrum of FAs was determined from stool samples. Since we also determined the FA composition of the food pellet, we confirmed that most of the determined FAs are not derived from the diet but are the result of gut bacterial metabolism.

On the other hand, this study has several limitations. First, we did not determine the gut microbiota present in fecal samples. Thus, our results only indirectly indicate the effect of gut microbiota on the FA composition of feces. Based on the results of previous highly extensive studies^31,32,35^ we can say that the ABx treatment we used also greatly reduced the intestinal microbiota^39^, which returned after the probiotic treatment. However, we cannot say which strains are responsible for the changes or whether there is a change in the composition of the microbiome before and after ABx and probiotics treatment. The use of a broad-spectrum treatment consisting of several types of ABx can also be a limiting factor, as different strains of gut bacteria react differently to various ABx^31,32^. However, the purpose of the present study was primarily to investigate the effect of a great reduction of gut microbiota, which was best accomplished with a broad-spectrum ABx mixture^39^. Thus, further systematic studies are necessary to understand more extensively the role of the microbiome on the FAs production and simultaneously deeper comprehending the relationship between these mechanisms and how they affect the physiological functions.

This is one of the first papers to analyze the role of gut bacteria on the detailed FA composition of stool samples from adult rats. The results demonstrated that, in addition to SCFAs, a number of other saturated and unsaturated FAs as well as *trans* and branched-chain saturated FAs are determined by gut bacteria in rat stool. To the best of our knowledge, this study is among the first ones to demonstrate that ABx treatment reduces the concentration of FAs in rat feces and that a specific probiotic mixture can restore this disturbed FA spectrum. Thus, this study not only provides evidence for the role of intestinal bacteria in the production of specific FAs, but it also supports a new approach to treat disorders resulting from a shift in the ratio and concentration of FAs. In conclusion, this study provides preliminary evidence that the gut microbiome appears to have a greater influence on both the quantitative and qualitative FA composition of feces than previously thought and that certain specific probiotic bacterial combinations are able to effectively restore the healthy feces conditions, indicating the potential therapeutic value of these probiotics.

## Methods

### Animals

In the present study, in total, 44 male Wistar laboratory rats were used (10 weeks old at the beginning of treatment)^36^. To exclude sex as an additional independent variable, only male rats were used in this research. Animals were kept individually in a temperature- and light-controlled room (12:12 h light–dark cycle; 21 ± 2 °C; humidity 55–60%). All experimental groups received the same laboratory food pellets (LT/R standard rodent food pellet, Innovo Kft, Isaszeg, Hungary) (Supplementary Table S5) and tap water ad libitum. The animals were cared for in accordance with the National Scientific Ethical Committee on Animal Experimentation of Hungary ((BA02/2000–07/2023 Pécs University, Medical School; Hungarian Government Decree, 40/2013. (II. 14.); NIH Guidelines, 1997; European Community Council Directive 86/609/EEC 1986, 2006; European Directive 2010/63/EU of the European Parliament). The present study is reported in accordance with ARRIVE guidelines.

The ABx treated groups were given broad-spectrum ABx mixture for 4 weeks (10 weeks old at the beginning of treatment) to greatly reduce the gut microbiota^39^. The ABx mixture was dissolved in their drinking water and comprised of ampicillin (1 g/L), vancomycin (500 mg/L), ciprofloxacin HCl (20 mg/L), imipenem (250 mg/L) and metronidazole (1 g/L). The probiotic receiving groups were given our specific probiotics mixture (PM) via oral gavage every day for 2 weeks. It contained four beneficial bacterial species (*Lactobacillus spp., Bifidobacterium spp.)* of specified cfu/d (colony forming units/day). This mixture is proprietary knowledge licensed by the University of Pécs (422.lbh.5.(2019.09.05)). The strains were provided by the Leibniz Institute, DSMZ-German Collection of Microorganisms and Cell Cultures GmbH, Germany. After the strains were received in freeze—dried form, we started to cultivate them. *Lactobacillus spp*. strains were cultivated in 100–100 mL liquid Rogosa medium (OXOID Ltd. UK) for 2 nights at 37 °C in a shaker incubator at 200 rpm. Then the fluids were centrifuged at 4 °C with 5.000 rpm for 5 min. The sediment was resuspended in physiological saline solution till reaching the final volume. *Bifidobacterium spp.* strains were cultivated on fastidious anaerobic agar CE plates and broth (Neogen Europe Ltd. UK). Anaerobic conditions were produced in an anaerobic jar with GEnbag anaer (BioMérieux SA France). After 2 days of cultivation, the colonies from the plates were collected by loop and resuspended in physiological saline solution till reaching the final volume. The prebiotic mixture was produced from day to day shortly before its use in experiment. More details about the cultivation can be found in our previous publication^36^. Throughout the whole experiment water and food consumption were measured every day. Fresh fecal pellets were collected under controlled conditions before and after the treatments to monitor the changes of the FAs. The samples were collected at three different times (before, during and after treatments) in each group (Fig. 1).

### Fatty acid analysis

The feces samples were analyzed by the slightly modified method of Lopez-Lopez^58^. From each sample, approximately 100 mg fecal content was placed in 16 × 125 mm test tubes sealed with Teflon-lined caps. We added 40 μl solution containing 60 mg methyl tridecanoate (C13:0) in 20 ml of n-hexane to each tube with the sample. We added 3 ml of methanol:hexane 4:1 (V/V) and 1–2 mg pyrogallol to the samples, then these samples were frozen for half an hour. 300 μl of acetyl chloride was slowly added to the frozen samples. The tubes were subjected to methanolysis at 100 °C for 1 h. After the tubes cooled down, 5 ml of 6% K_2_CO_3_ solution was slowly added to stop the reaction and to neutralize the mixture. The tubes were vortex-mixed and centrifuged at 3000 × g for 10 min. The supernatant hexane layer was then transferred to a 2 ml screw-top vial with a micropipette. After this step, hexane was added until reaching the liquid level of 1 ml. The samples were stored in a −80 °C freezer until injection into the gas chromatograph (GC).

The FA analyses were carried out on a PerkinElmer Clarus 690 gas chromatograph with a flame ionization detector (PerkinElmer, USA) fitted with a Rt-2560 capillary column (100 m × 0.25 mm i.d. × 0.20 µm film thickness, Restek). Helium was used as the carrier gas at a flow rate of 1.3 ml/min. The injection port was adjusted at 225 °C and split injection mode was used, the injection ratio was 20:1. The injection volume was 2 µl. The detector temperature was 300 °C. The initial oven temperature was 100 ℃ held for 4 min and ramped up to 250 °C at 3 °C/min and held for 25 min.

The fatty acid methyl esters (FAMEs) were analyzed based on the area under the curve calculated using TotalChrom software by PerkinElmer. Fatty acid identification was based on the retention times of external standards (PUFA3 (Supelco, St. Louis, MO, USA); GLC-674, -642, -643, -569b, -481, and -473 (Nu-Check-Prep, Elysian, MN, USA); C16:1n-9-ME (Larodan AB, Solna, Sweden) and The Bacterial Acid Methyl Esters CP Mixture (Matreya LLC., State College, PA, USA), Supplementary Table S6). The peaks were identified by comparing them with authentic mixtures of the weighed FAME methyl ester. The individual FA response factors determined from these weighed standards and the percentage area under the curve (relative concentration; w/w%) were used to calculate the weight percentage of each determined FA^59^. The quantification of the FA concentrations of wet feces was based on the tridecanoic acid, as internal standard (absolute concentration; μg FA /100 mg wet feces).

### Statistics

For classifying the sample’s GC chromatograms Principal Component Analysis (PCA) was applied. The input data for the PCA was the area under the curve of the components. PCA transforms data into a lower dimensional space by converting the data to a new, orthogonal coordinate system called principal components (PCs). PCs are sorted according to the amount of variance captured, in such a way that those that have similar variance occur near one another. If the chemical variations are similar, their scores should be close to each other on a plot of PC1 against PC2. The PCA model was fitted using Eigen value decomposition method.

To detect the effect of the treatments in time on the FA composition and concentration we applied a nested linear mixed random effect model, where the ID of the rats were used as a random factor and the nested effect was the litter. In case of modeling the changes in the concentrations we used log transformation to ensure a proper model fit. In all models we used the time point of the sample taking, treatments and their interaction as explanatory factors and the models were adjusted to the baseline values. The input data for the mixed model were the calculated concentration on the log scale and the calculated percentage of FAs. The models were fitted by using the Restricted Maximum Likelihood (REML) approach and the estimates were given with the Satterthwaite's method.

All analyses were done with R statistical software (version 4.2.1; packages:lme4, lmerTest; R Core Team, Vienna, Austria)^60,61^. A result was set as significant if the *p* value was under 0.05.

### Ethical approval

All animal experiments were conducted according to federal and local ethical guidelines, and the protocols were approved by the National Scientific Ethical Committee on Animal Experimentation of Hungary ((BA02/2000–07/2023, Pécs University, Medical School; Hungarian Government Decree, 40/2013. (II. 14.); NIH Guidelines, 1997; European Community Council Directive 86/609/EEC 1986, 2006; European Directive 2010/63/EU of the European Parliament). The present study is reported in accordance with ARRIVE guidelines.

### Consent for publication

All authors have read the manuscript and approved to be co-authors on the manuscript and have substantial contribution in the manuscript.

### Supplementary Information

Supplementary Information 1.

Supplementary Information 2.

Supplementary Information 3.

## Data Availability

We confirm that all information is included in the manuscript or in the supplementary information files.

## Abbreviations

ABx: Antibiotics
ALA: Alpha-linolenic acid
CNS: Central nervous system
FA: Fatty acids
FAME: Fatty acid methyl ester
GC: Gas chromatograph
GI: Gastrointestinal
LCPUFA: Long chain polyunsaturated fatty acid
MUFA: Monounsaturated fatty acid
PCA: Principal component analysis
PCs: Principal components
PM: Probiotic mixture
PUFAs: Polyunsaturated fatty acids
REML: Restricted maximum likelihood
SAT: Saturated fatty acids
SCFAs: Short-chain fatty acids

## References

[1] Zárate R, Jaber-Vazdekis N, Tejera N, Pérez JA, Rodríguez C (2017). Significance of long chain polyunsaturated fatty acids in human health. Clin. Trans. Med..

[2] Rustan, A. C. & Drevon, C. A. Fatty Acids: Structures and Properties. In *Encyclopedia of Life Sciences* (John Wiley and Sons, Ltd., Chichester, 2005). 10.1038/npg.els.0003894.

[3] Taormina VM, Unger AL, Schiksnis MR, Torres-Gonzalez M, Kraft J (2020). Branched-chain fatty acids—an underexplored class of dairy-derived fatty acids. Nutrients.

[4] Nakamura MT, Yudell BE, Loor JJ (2014). Regulation of energy metabolism by long-chain fatty acids. Prog. Lipid Res..

[5] Flock MR, Kris-Etherton PM (2013). Diverse physiological effects of long-chain saturated fatty acids. Curr. Opin. Clin. Nutr. Metab. Care.

[6] Baker EJ, Yusof MH, Yaqoob P, Miles EA, Calder PC (2018). Omega-3 fatty acids and leukocyte-endothelium adhesion: Novel anti-atherosclerotic actions. Mol. Aspects Med..

[7] de Bus I, Witkamp R, Zuilhof H, Albada B, Balvers M (2019). The role of n-3 PUFA-derived fatty acid derivatives and their oxygenated metabolites in the modulation of inflammation. Prostaglandins Other Lipid Med..

[8] Kuziel GA, Rakoff-Nahoum S (2022). The gut microbiome. Curr. Biol..

[9] Fu Y (2021). Associations among dietary omega-3 polyunsaturated fatty acids, the gut microbiota, and intestinal immunity. Mediators Inflamm..

[10] Robertson RC (2018). Maternal omega-3 fatty acids regulate offspring obesity through persistent modulation of gut microbiota. Microbiome.

[11] Patterson E (2014). Impact of dietary fatty acids on metabolic activity and host intestinal microbiota composition in C57BL/6J mice. Br. J. Nutr..

[12] Zhuang P (2020). Eicosapentaenoic and docosahexaenoic acids differentially alter gut microbiome and reverse high-fat diet-induced insulin resistance. Mol. Nutr. Food Res..

[13] Wood KE, Mantzioris E, Gibson RA, Ramsden CE, Muhlhausler BS (2015). The effect of modifying dietary LA and ALA intakes on omega-3 long chain polyunsaturated fatty acid (n-3 LCPUFA) status in human adults: A systematic review and commentary. Prostaglandins Leukot. Essent. Fatty Acids.

[14] Dahl, W. J., Rivero Mendoza, D. & Lambert, J. M. Diet, nutrients and the microbiome. In *The Microbiome in Health and Disease Progress in Molecular Biology and Translational Science* 237–263 (2020). 10.1016/bs.pmbts.2020.04.006. 10.1016/bs.pmbts.2020.04.006 32475524

[15] O'Shea EF, Cotter PD, Stanton C, Ross RP, Hill C (2012). Production of bioactive substances by intestinal bacteria as a basis for explaining probiotic mechanisms: Bacteriocins and conjugated linoleic acid. Int. J. Food Microbiol..

[16] Wall R (2009). Metabolic activity of the enteric microbiota influences the fatty acid composition of murine and porcine liver and adipose tissues. Am. J. Clin. Nutr..

[17] Druart C (2014). Role of the lower and upper intestine in the production and absorption of gut microbiota-derived PUFA metabolites. PLoS ONE.

[18] Zhao L (2018). Saturated long-chain fatty acid-producing bacteria contribute to enhanced colonic motility in rats. Microbiome.

[19] Peng M, Biswas D (2016). Short chain and polyunsaturated fatty acids in host gut health and foodborne bacterial pathogen inhibition. Crit. Rev. Food Sci. Nutr..

[20] Alexander C, Swanson KS, Fahey GC, Garleb KA (2019). Perspective: Physiologic importance of short-chain fatty acids from nondigestible carbohydrate fermentation. Adv. Nutr..

[21] Smith EA, Macfarlane GT (1997). Dissimilatory amino Acid metabolism in human colonic bacteria. Anaerobe.

[22] Koh A, De Vadder F, Kovatcheva-Datchary P, Bäckhed F (2016). From dietary fiber to host physiology: Short-chain fatty acids as key bacterial metabolites. Cell.

[23] Holscher HD (2017). Dietary fiber and prebiotics and the gastrointestinal microbiota. Gut Microbes.

[24] Wang H-X, Wang Y-P (2016). Gut Microbiota-brain Axis. Chin. Med. J..

[25] Silva YP, Bernardi A, Frozza RL (2020). The role of short-chain fatty acids from gut microbiota in gut-brain communication. Front. Endocrinol..

[26] Mayer EA, Knight R, Mazmanian SK, Cryan JF, Tillisch K (2014). Gut microbes and the brain: Paradigm shift in neuroscience. J. Neurosci..

[27] Lee KH, Song Y, Wu W, Yu K, Zhang G (2020). The gut microbiota, environmental factors, and links to the development of food allergy. Clin. Mol. Allergy.

[28] Gomaa EZ (2020). Human gut microbiota/microbiome in health and diseases: a review. Antonie van Leeuwenhoek.

[29] Sasmita AO (2019). Modification of the gut microbiome to combat neurodegeneration. Rev. Neurosci..

[30] Pandey KR, Naik SR, Vakil BV (2015). Probiotics, prebiotics and synbiotics—-a review. J. Food Sci. Technol..

[31] Langdon A, Crook N, Dantas G (2016). The effects of antibiotics on the microbiome throughout development and alternative approaches for therapeutic modulation. Genome Med..

[32] Iizumi, T., Battaglia, T., Ruiz, V. & Perez Perez, G. I. Gut Microbiome and Antibiotics. *Arch. Med. Res.* **48**, 727–734, 10.1016/j.arcmed.2017.11.004 (2017). 10.1016/j.arcmed.2017.11.004 29221800

[33] Moloney RD, Desbonnet L, Clarke G, Dinan TG, Cryan JF (2013). The microbiome: stress, health and disease. Mammalian Genome.

[34] Zhao X (2016). Sensitive and simplified detection of antibiotic influence on the dynamic and versatile changes of fecal short-chain fatty acids. Plos One.

[35] Pauline M (2022). Probiotic treatment vs empiric oral antibiotics for managing dysbiosis in short bowel syndrome: Impact on the mucosal and stool microbiota, short-chain fatty acids, and adaptation. J Parenter. Enteral Nutr..

[36] Mintál K (2022). Novel probiotic treatment of autism spectrum disorder associated social behavioral symptoms in two rodent models. Sci. Rep..

[37] Kao, A. C. C., Harty, S. & Burnet, P. W. J. in *Gut Microbiome and Behavior International Review of Neurobiology* 21–48 (2016). 10.1016/bs.irn.2016.08.007 27793220

[38] Borre, Y. E., Moloney, R. D., Clarke, G., Dinan, T. G. & Cryan, J. F. The Impact of Microbiota on Brain and Behavior: Mechanisms & Therapeutic Potential. In *Microbial Endocrinology: The Microbiota-Gut-Brain Axis in Health and Disease Advances in Experimental Medicine and Biology* Ch. 17, 373–403 (2014). 10.1007/978-1-4939-0897-4_17. 10.1007/978-1-4939-0897-4_17 24997043

[39] Hoban AE (2016). Behavioural and neurochemical consequences of chronic gut microbiota depletion during adulthood in the rat. Neuroscience.

[40] Babu AKS (2024). Gut microbiota depletion using antibiotics to investigate diet-derived microbial metabolites: An efficient strategy. Mol. Nutr. Food Res..

[41] Yuille S, Reichardt N, Panda S, Dunbar H, Mulder IE (2018). Human gut bacteria as potent class I histone deacetylase inhibitors in vitro through production of butyric acid and valeric acid. Plos One.

[42] Chen R (2019). Transplantation of fecal microbiota rich in short chain fatty acids and butyric acid treat cerebral ischemic stroke by regulating gut microbiota. Pharmacol. Res..

[43] Trefflich I, Dietrich S, Braune A, Abraham K, Weikert C (2021). Short- and branched-chain fatty acids as fecal markers for microbiota activity in vegans and omnivores. Nutrients.

[44] Choi BSY (2021). Feeding diversified protein sources exacerbates hepatic insulin resistance via increased gut microbial branched-chain fatty acids and mTORC1 signaling in obese mice. Nat. Commun..

[45] Gozdzik, P., Magkos, F., Sledzinski, T. & Mika, A. Monomethyl branched-chain fatty acids: Health effects and biological mechanisms. *Progress in Lipid Research* **90**, ARTN 101226 10.1016/j.plipres.2023.101226 (2023). 10.1016/j.plipres.2023.101226 37094753

[46] Schuchardt JP, Huss M, Stauss-Grabo M, Hahn A (2009). Significance of long-chain polyunsaturated fatty acids (PUFAs) for the development and behaviour of children. Eur. J. Pediatrics.

[47] Kulkarni A, Zhao A, Yang B, Zhang Y, Linderborg KM (2022). Foods.

[48] Di Rienzi SC (2021). The microbiome affects liver sphingolipids and plasma fatty acids in a murine model of the Western diet based on soybean oil. J. Nutr. Biochem..

[49] Albouery M (2021). Soluble fiber inulin consumption limits alterations of the gut microbiota and hepatic fatty acid metabolism caused by high-fat diet. Nutrients.

[50] Xu C (2021). Multi-omics analysis reveals a dependent relationship between rumen bacteria and diet of grass- and grain-fed yaks. Front. Microbiol..

[51] Aghamohammad, S. *et al.* Anti‐inflammatory and immunomodulatory effects of Lactobacillus spp. as a preservative and therapeutic agent for IBD control. *Immunity, Inflammation and Disease* **10**, 10.1002/iid3.635 (2022). 10.1002/iid3.635 PMC9119005 35634951

[52] Kaźmierczak-Siedlecka K, Roviello G, Catalano M, Polom K (2021). Gut microbiota modulation in the context of immune-related aspects of Lactobacillus spp. and Bifidobacterium spp. Gastrointestinal Cancers. Nutrients.

[53] Ng, Q. X. *et al.* Use of Lactobacillus spp. to prevent recurrent urinary tract infections in females. *Med. Hypotheses* **114**, 49–54, 10.1016/j.mehy.2018.03.001 (2018). 10.1016/j.mehy.2018.03.001 29602464

[54] Cui S, Hu Y (2012). Multistrain probiotic preparation significantly reduces symptoms of irritable bowel syndrome in a double-blind placebo-controlled study. Int. J. Clin. Exp. Med..

[55] He B-L, Xiong Y, Hu T-G, Zong M-H, Wu H (2022). Bifidobacterium spp. as functional foods: A review of current status, challenges, and strategies. Crit. Rev. Food Sci. Nutr..

[56] Zhang D (2019). Fecal microbiota and its correlation with fatty acids and free amino acids metabolism in piglets after a lactobacillus strain oral administration. Front. Microbiol..

[57] Huang Y, Jiao J, Yao D, Guo F, Li Y (2023). Altered fecal microbiome and metabolome profiles in rat models of short bowel syndrome. Front. Microbiol..

[58] López-López A, Castellote-Bargalló AI, López-Sabater MC (2000). Comparison of two direct methods for the determination of fatty acids in infant feces. Anal. Biochem..

[59] Szabó É (2007). trans Octadecenoic acid and trans octadecadienoic acid are inversely related to long-chain polyunsaturates in human milk: results of a large birth cohort study. Am. J. Clin. Nutr..

[60] Kuznetsova, A., Brockhoff, P. B. & Christensen, R. H. B. lmerTest Package: Tests in linear mixed effects models. *J. Stat. Softw.* **82**, 10.18637/jss.v082.i13 (2017).

[61] Bates, D., Mächler, M., Bolker, B. & Walker, S. Fitting linear mixed-effects models Usinglme4. *J. Stat. Softw.* **67**, 10.18637/jss.v067.i01 (2015).

