# Supplementary material for: Antibiotics and probiotics-induced effects on the total fatty acid composition of feces in a rat model
Source: Sci Rep. 2024 Mar 19;14:6542. doi: 10.1038/s41598-024-57046-6 (PMC10951306; doi:10.1038/s41598-024-57046-6)
Supplement: Supplementary file 3 — Supplementary Information 3. [file 41598_2024_57046_MOESM3_ESM.pdf]

Supplementary files

Antibiotics and probiotics-induced effects on the total fatty acid composition of feces in a rat model

Tamás Marosvölgyi[1,+], Kitti Mintál[2,3,+], Nelli Farkas[1], Zoltán Sipos[1], Lilla Makszin[1], Éva Szabó[4,*], Attila Tóth[2,3], Béla Kocsis[5], Krisztina Kovács[5], Edina Hormay[2,3], László Lénárd[2,3], Zoltán Karádi[2,3], Anita Bufa[1]

[1]Institute of Bioanalysis, Medical School, University of Pécs, Pécs, 7624, Hungary

[2]Institute of Physiology, Medical School, University of Pécs, Pécs, 7624, Hungary

[3]Medical and Engineering Multidisciplinary Cellular Bioimpedance Research Group, Szentágothai Research Centre, University of Pécs, Pécs, 7624, Hungary

[4]Department of Biochemistry and Medical Chemistry, Medical School, University of Pécs, Pécs, 7624, Hungary

[5]Department of Medical Microbiology and Immunology, Medical School, University of Pécs, Pécs, 7624, Hungary

[*]szabo.eva.dr@pte.hu

Supplementary Table S1: Results of the mixed effect models of the determined fatty acids for both concentration and weight percent values.

Fatty acid	The nested linear mixed effect model					
	p value for concentrations			p value for weight%		
	time point	treatment	time point x treatment	time point	treatment	time point x treatment
C4:0	**4.17×10^{-13}**	**3.70×10^{-9}**	**2.15×10^{-10}**	**4.11×10^{-7}**	**3.18×10^{-7}**	**1.15×10^{-3}**
C5:0	**1.41×10^{-6}**	**1.83×10^{-13}**	**4.96×10^{-5}**	**1.35×10^{-2}**	**1.54×10^{-2}**	0.111
C6:0	**9.52×10^{-3}**	**2.32×10^{-9}**	0.201	0.0639	**1.53×10^{-4}**	0.389
C7:0	0.474	**1.51×10^{-3}**	**4.12×10^{-2}**	0.953	0.272	0.130
C8:0	**1.43×10^{-2}**	**2.76×10^{-4}**	0.733	0.0559	**1.90×10^{-3}**	0.858
C9:0	0.498	0.145	0.275	0.327	0.0966	0.271
C10:0	**2.15×10^{-2}**	**5.04×10^{-12}**	0.0532	**3.68×10^{-2}**	**2.54×10^{-10}**	0.0575
C11:0	0.433	**3.35×10^{-10}**	0.619	0.286	**1.44×10^{-10}**	0.316
C12:0	**1.45×10^{-6}**	**1.09×10^{-7}**	**8.91×10^{-4}**	0.695	**1.42×10^{-2}**	0.998
C13ai	**2.92×10^{-6}**	**$< 2.2 \times 10^{-16}$**	**2.77×10^{-7}**	**1.31×10^{-6}**	**2.39×10^{-13}**	**7.03×10^{-8}**
C14i	**3.16×10^{-16}**	**$< 2.2 \times 10^{-16}$**	**$< 2.2 \times 10^{-16}$**	**4.71×10^{-15}**	**$< 2.2 \times 10^{-16}$**	**$< 2.2 \times 10^{-16}$**
C13:1n-1	**6.97×10^{-14}**	**$< 2.2 \times 10^{-16}$**	**$< 2.2 \times 10^{-16}$**	**9.67×10^{-11}**	**$< 2.2 \times 10^{-16}$**	**$< 2.2 \times 10^{-16}$**
C14:0	**6.92×10^{-11}**	**3.52×10^{-11}**	**8.24×10^{-12}**	**9.44×10^{-6}**	**3.39×10^{-8}**	**3.55×10^{-6}**
C15i	**4.82×10^{-15}**	**$< 2.2 \times 10^{-16}$**	**$< 2.2 \times 10^{-16}$**	**6.10×10^{-12}**	**$< 2.2 \times 10^{-16}$**	**$< 2.2 \times 10^{-16}$**
C15ai	**8.94×10^{-11}**	**$< 2.2 \times 10^{-16}$**	**$< 2.2 \times 10^{-16}$**	**3.31×10^{-7}**	**$< 2.2 \times 10^{-16}$**	**1.07×10^{-14}**
C14:1n-5	**1.67×10^{-5}**	0.62	**2.29×10^{-5}**	**1.16×10^{-4}**	0.853	**1.03×10^{-4}**
C15:0	**1.73×10^{-11}**	**$< 2.2 \times 10^{-16}$**	**1.93×10^{-15}**	**9.45×10^{-10}**	**$< 2.2 \times 10^{-16}$**	**$< 2.2 \times 10^{-16}$**
C16i	**5.34×10^{-6}**	**2.93×10^{-15}**	**4.22×10^{-10}**	**4.49×10^{-2}**	**4.22×10^{-8}**	**9.59×10^{-5}**
C16:0	**2.59×10^{-3}**	**3.31×10^{-7}**	**6.62×10^{-5}**	0.505	0.898	0.154
tC16:1n-7+C17i	**3.13×10^{-4}**	**2.26×10^{-9}**	**4.51×10^{-7}**	0.135	**7.06×10^{-6}**	**3.11×10^{-2}**
C16:1n-9	0.129	**4.91×10^{-7}**	**3.96×10^{-2}**	0.690	**3.57×10^{-2}**	0.650

**C16:1n-7**	**3.25×10^{-4}**	**7.49×10^{-6}**	**1.40×10^{-2}**	0.247	0.0956	0.843
**C17:0**	**1.01×10^{-4}**	**6.77×10^{-14}**	**2.77×10^{-7}**	0.0969	**3.16×10^{-6}**	**4.02×10^{-3}**
**tC17:1n-7+C18i**	**3.41×10^{-4}**	**8.79×10^{-8}**	**5.32×10^{-7}**	0.785	0.0695	**2.07×10^{-2}**
**C18:0**	**2.08×10^{-4}**	**2.52×10^{-12}**	**2.59×10^{-7}**	0.478	**9.08×10^{-5}**	**4.27×10^{-4}**
**t18:1 mix**	**5.82×10^{-7}**	**$< 2.2 \times 10^{-16}$**	**3.55×10^{-14}**	**2.53×10^{-3}**	**7.60×10^{-13}**	**8.06×10^{-8}**
**C18:1n-9**	**1.96×10^{-3}**	**2.50×10^{-6}**	**5.33×10^{-3}**	**3.82×10^{-3}**	**3.11×10^{-6}**	**1.42×10^{-3}**
**C18:1n-7**	**3.43×10^{-5}**	**2.11×10^{-8}**	**3.78×10^{-4}**	0.102	**1.82×10^{-3}**	0.544
**C18:2n-6**	**2.41×10^{-2}**	0.0524	0.242	**5.23×10^{-4}**	**1.09×10^{-14}**	**3.00×10^{-7}**
**C20:0**	**2.71×10^{-4}**	**1.10×10^{-8}**	**1.85×10^{-6}**	0.450	0.0524	**3.68×10^{-4}**
**C20:1n-9**	**1.07×10^{-4}**	**1.63×10^{-8}**	**1.53×10^{-5}**	0.861	0.176	0.101
**C18:3n-3**	**5.00×10^{-2}**	**1.53×10^{-2}**	0.217	**1.62×10^{-4}**	**2.14×10^{-14}**	**1.05×10^{-5}**
**C20:2n-6**	**2.25×10^{-6}**	**8.52×10^{-7}**	**3.17×10^{-6}**	0.987	0.282	0.797
**C22:0**	**3.68×10^{-3}**	**1.56×10^{-8}**	**3.32×10^{-4}**	0.155	0.275	0.881
**C20:3n-6**	**3.35×10^{-3}**	**1.71×10^{-4}**	**8.77×10^{-3}**	0.630	0.290	0.823
**C22:1n-9**	**4.81×10^{-3}**	**1.36×10^{-5}**	0.0962	0.246	0.647	0.866
**C23:0**	**2.19×10^{-3}**	**2.34×10^{-4}**	**1.03×10^{-3}**	0.365	0.363	0.380
**C20:4n-6**	0.734	0.0918	0.316	**3.46×10^{-7}**	**9.08×10^{-7}**	**1.90×10^{-10}**
**C24:0**	**1.21×10^{-3}**	**2.62×10^{-8}**	**7.40×10^{-05}**	0.335	0.104	0.429
**C20:5n-3**	0.534	0.284	0.795	0.916	0.560	0.915
**C25:0**	0.493	**4.16×10^{-2}**	0.0545	0.500	0.578	0.593
**C22:4n-6**	0.164	**3.93×10^{-2}**	0.641	0.657	0.0666	0.955
**C22:5n-6**	**1.24×10^{-2}**	**2.17×10^{-3}**	0.263	0.734	0.714	**3.10×10^{-2}**
**C26:0**	**5.76×10^{-3}**	**2.58×10^{-7}**	**4.89×10^{-4}**	**1.19×10^{-2}**	**8.84×10^{-3}**	0.354

Significances are represented by bold characters ($p < 0.05$).

Supplementary Table S2: Fatty acid concentration (μg fatty acid / 100 mg wet stool) values in the four treatment groups at each sampling time.

See SupplementaryTableS2.xlsx

Supplementary Table S3: Fatty acid weight percent (w/w%) values in the four treatment groups at each sampling time.

See SupplementaryTableS3.xlsx

Supplementary Table S4: Fatty acid composition of the LT/R standard rodent food pellet by Innovo Ltd.

w/w %	Mean	SD
**C14:0**	0.31	0.01
**C16:0**	22.76	0.07
**C18:0**	5.34	0.03
**t18:1mix**	0.09	0.01
**C18:1n-9**	34.05	0.14
**C18:1n-7**	1.29	0.01
**C18:2n-6**	29.31	0.21
**C18:3n-3**	1.40	0.03
**N-3 PUFA**	1.52	0.03
**N-3 LCPUFA**	0.12	0.01
**n-6 PUFA**	29.64	0.21
**N-6 LCPUFA**	0.33	0.00
**PUFA**	31.16	0.23
**LCPUFA**	0.45	0.01
**MUFA**	36.09	0.14
**SAT**	27.32	0.18
**Trans**	0.09	0.01
**Branched sat**	-	-
**Trans+Branched all**	0.10	0.01
**short SAT**	1.37	0.19

Mean (SD) values are based on 3 parallel chemical analysis.

Supplementary Table S5: Composition and energy content of the LT/R standard rodent food pellet by Innovo Ltd

Ingredients	maize, wheat, barley, sunflower meal, soya meal, milk powder, yeast, lime, feed meal, sunflower oil, premix
Dry matter	82.1 %
Crude protein	20.1 %
Crude fat	3.9 %
Crude fibre	4.5 %
Crude ash	7.8 %
Metab. energy	11.9 MJ/kg

Supplementary Table S6: Retention times of determined fatty acids in the used external standards

Retention time (min)	FAME	GLC463	GLC473b	GLC481	GLC569b	GLC674	GLC642	GLC643	C16:1n-9	BAME CP MIX 1114	Alles
10.64	C4:0	*			*	*					*
11.51	C5:0	*									
12.69	C6:0	*			*	*					*
14.25	C7:0	*									
16.22	C8:0	*			*	*					*
18.51	C9:0	*									
21.04	C10:0	*	*		*	*					*
23.65	C11:0	*			*	*				*	*
26.26	C12:0	*	*		*	*				*	*
28.11	C13ai										*
28.83	C13:0	*			*	*				*	*
30.05	C14i										*
31.01	C13:1n-1	*									
31.30	C14:0	*	*		*	*				*	*
32.49	C15i									*	
32.96	C15ai									*	*
33.27	C14:1n-5	*	*		*	*					*
33.68	C15:0	*			*	*				*	*
34.81	C16i									*	*
35.99	C16:0	*	*		*	*				*	*
37.04	tC16:1n-7	*	*	*	*	*					*
37.04	C17i									*	
37.31	C16:1n-9								*		
37.53	C16:1n-7	*	*		*	*					*
38.13	C17:0	*	*		*	*				*	*
39.17	tC17:1n-7			*	*	*					*
39.17	C18i										*
40.24	C18:0	*	*		*	*				*	*
41.08	t18:1mix	*	*	*	*	*					*
41.55	C18:1n-9	*	*		*	*					*
41.72	C18:1n-7	*	*								*
43.49	C18:2n-6	*	*		*	*		*			*
44.13	C20:0	*	*		*	*				*	*
45.36	C20:1n-9	*	*		*	*					*
45.63	C18:3n-3	*	*		*	*	*				*
47.17	C20:2n-6	*	*		*	*		*			*
47.75	C22:0	*	*		*	*					*
48.51	C20:3n-6	*	*		*	*		*			*
48.93	C22:1n-9	*	*		*	*					*

49.46	C23:0		*		*	*					*
49.52	C20:4n-6	*	*		*	*		*			*
51.13	C24:0	*	*		*	*					*
51.67	C20:5n-3	*	*		*	*	*				*
52.73	C25:0		*								
53.14	C22:4n-6	*	*		*	*		*			*
53.92	C22:5n-6				*	*		*			*
54.29	C26:0		*								

The asterisks denote which fatty acid was present in the given standards.

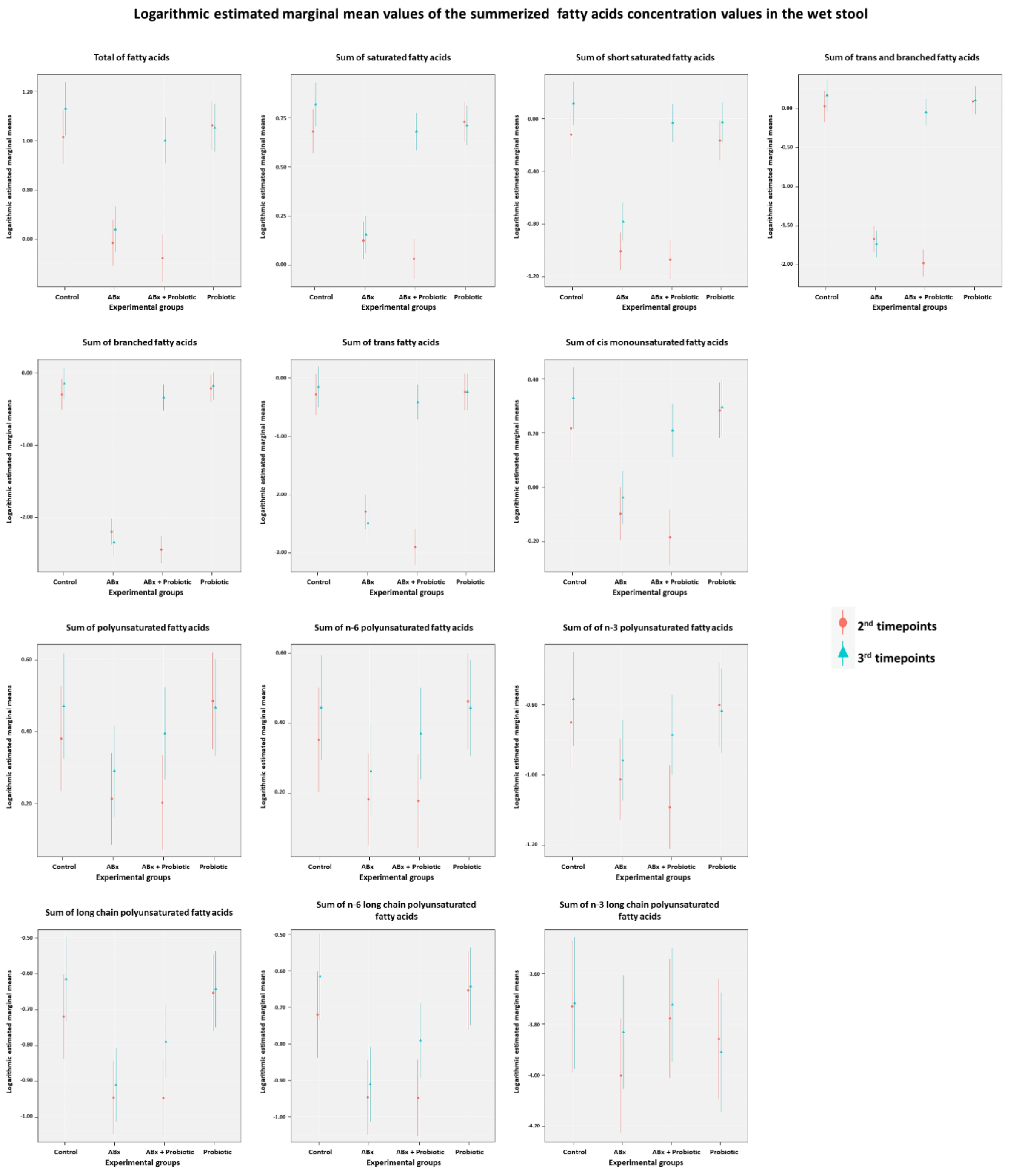

Supplementary Figure S1: Logarithmic estimated marginal mean values of the summarized fatty acid concentrations (in µg/100 mg wet feces) at the 2nd and 3rd sampling times in the four treatment groups

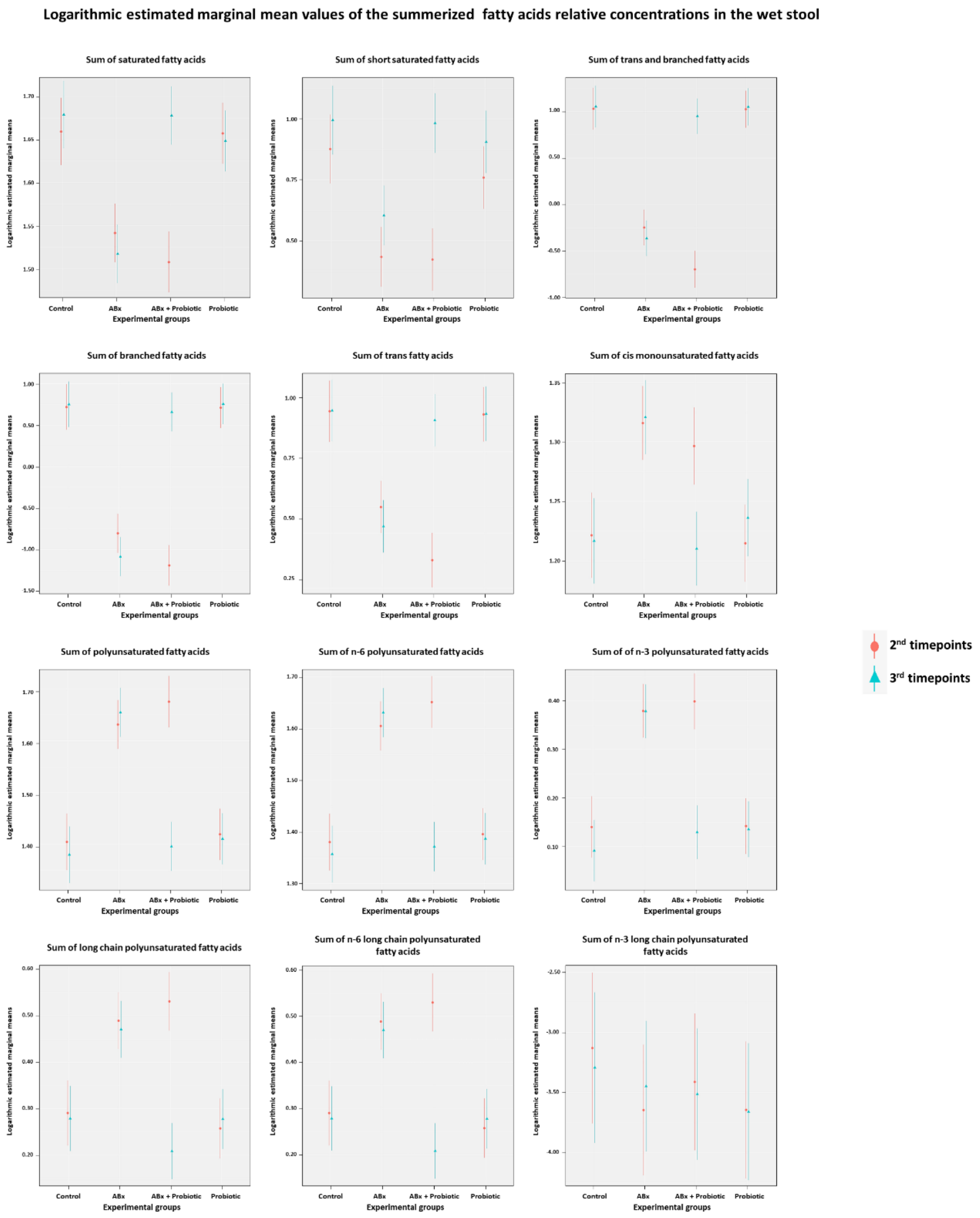

Supplementary Figure S2: Logarithmic estimated marginal mean values of the summarized fatty acid weight percent ratios at the 2nd and 3rd sampling times in the four treatment groups
